# Research progress on mesenchymal stem cells and their exosomes in systemic sclerosis

**DOI:** 10.3389/fphar.2023.1263839

**Published:** 2023-08-25

**Authors:** Yan Zhang, Yanli Yang, Xiang Gao, Wenqin Gao, Liyun Zhang

**Affiliations:** Third Hospital of Shanxi Medical University, Shanxi Bethune Hospital, Shanxi Academy of Medical Sciences, Tongji Shanxi Hospital, Taiyuan, China

**Keywords:** mesenchymal stem cells, exosomes, systemic sclerosis, pulmonary interstitial fibrosis, skin fibrosis, vascular injury, other organ involvement

## Abstract

Systemic sclerosis (SSc) is a connective tissue disease with an unknown etiology. Clinically, it is characterized by localized or diffuse skin thickening and fibrosis. The pathogenesis of SSc includes microvascular injury, autoimmune-mediated inflammation, and fibroblast activation. These processes interact and contribute to the diverse clinicopathology and presentation of SSc. Given the limited effectiveness and substantial side effects of traditional treatments, the treatment strategy for SSc has several disadvantages. Mesenchymal stem cells (MSCs) are expected to serve as effective treatment options owing to their significant immunomodulatory, antifibrotic, and pro-angiogenic effects. Exosomes, secreted by MSCs via paracrine signaling, mirror the effect of MSCs as well as offer the benefit of targeted delivery, minimal immunogenicity, robust reparability, good safety and stability, and easy storage and transport. This enables them to circumvent the limitations of the MSCs. When using exosomes, it is crucial to consider preparation methods, quality standards, and suitable drug delivery systems, among other technical issues. Therefore, this review aims to summarize the latest research progress on MSCs and exosomes in SSc, offering novel ideas for treating SSc.

## 1 Introduction

Systemic sclerosis (SSc) is a type of autoimmune connective tissue disease involving multiple organs and characterized by skin fibrosis. In this disease condition, autoantibodies and collagen accumulation compromise the normal tissue architecture, leading to skin fibrosis and multiple organ dysfunction (Tamby et al., 2003; Denton and Black, 2004; Servettaz et al., 2006). Furthermore, despite a prevalence ranging from 50 to 300 cases per million and a predilection for individuals aged 30–50 years, the standardized mortality rate of SSc has shown insignificant change over the last 40 years, with one-third of patients dying within 10 years of disease diagnosis (Rubio-Rivas et al., 2014). Interstitial lung disease is the most common cause of death in patients with SSc. Previously, numerous therapy alternatives, including glucocorticoids, immunosuppressants, and antifibrotic medicines, have been utilized for symptom management and disease progression inhibition, yielding unsatisfactory outcomes. Autologous hematopoietic stem cell transplantation (AHSCT) emerged for treating refractory SSc in the past decade. This approach improves the degree of skin sclerosis and visceral lesions and significantly augments the overall survival of patients with SSc. The requirement for immunosuppressive medications before AHSCT, with concerns about transplantation-related mortality, introduces risks of infection, making broad adoption of AHSCT challenging (Turse et al., 2018). Recent studies have found a trend toward enhanced Forced Vital Capacity in patients with SSc treated with the biological drug Romilkimab compared with those not treated with it. However, phase III trials are needed to confirm these findings (Allanore et al., 2020). Consequently, there is an urgent need for medications with high efficacy and minimal adverse effects.

When activated by various inflammatory stimuli and cells, mesenchymal stem cells (MSCs) can migrate to the damaged location through blood arteries. They can perform anti-inflammatory, antifibrotic, and tissue repair functions by secreting soluble substances and extracellular vesicles. MSCs and their exosomes also have immunomodulatory properties. Exosomes, which are extracellular vesicles, serve a crucial function in parental cells and are carriers of a number of bioactive substances, making them more effective therapeutically (Cras et al., 2015). The potential of MSCs to treat diseases is being substantiated as researchers focus more on them. Furthermore, they are becoming increasingly essential for tissue engineering, tissue organ transplantation, gene therapy, and immunotherapy. Therefore, this review aims to outline the recent advancements in MSCs and their exosomes in systemic sclerosis. The findings of this study serve to provide a reference point for guiding future clinical research in treating patients with SSc.

## 2 Overview of MSCs and their exosomes

MSCs are adnexal, heterogeneous cells obtained from multiple organs with multidirectional differentiation potential (Friedenstein et al., 1976; Pittenger et al., 1999; Reinders et al., 2010; Ding et al., 2011; Shi et al., 2012). MSCs exhibit positive surface expression of CD73, CD90, and CD105 but negative surface expression of CD11b, CD14, CD19, CD79a, CD34, and CD45 (Dominici et al., 2006). MSCs are commonly used to treat immune system illnesses due to their non-rejection, self-healing, and immunomodulatory properties.

Exosomes are membrane nanovesicles derived from endosomes and range from 30 to 150 nm in size. They are secreted by all living cells and typically contain different biomolecules, such as proteins, lipids, cytokines, and RNAs (Cras et al., 2015; van Rhijn-Brouwer et al., 2016), which facilitate cell-to-cell signaling. MSC-derived exosomes include mRNAs, miRNAs, cytokines, and growth factors (Soundara et al., 2017). They have garnered much attention due to their lower immunogenicity than MSCs, coupled with their reduced risk of tumor formation (Moghadasi et al., 2021).

## 3 Pathogenesis of SSc

The pathogenesis of SSc primarily resolves around immune dysfunction (Dumoitier et al., 2014; Ganesan et al., 2023). This dysfunction disrupts B-cell homeostasis, thereby triggering autoantibody production and profibrotic cytokine secretion, such as interleukin-6 (IL-6) and interleukin-12 (IL-21) (FForestier et al., 2018). Furthermore, T-cell and specific helper T (Th cells) cells are essential for SSc pathophysiology. Profibrotic cytokines, specifically interleukin-13 and interleukin-4 (IL-4), are generated by Th2 cells, which are important in fibroblast activation and differentiation to myofibroblasts (Postlethwaite et al., 1992; Berlow et al., 2018). Additionally, Th17 cells exert pro-inflammatory effects. Nevertheless, the underlying mechanism through which they promote fibrosis remains partially understood (Xing et al., 2013; Liu et al., 2014; Liu et al., 2020; Xing et al., 2020). The pro-inflammatory cytokine chemokine (C-X-C motif) ligand 2 (CXCL 2) and collagen deposition have been associated with the profibrotic action of IL-17 A on fibroblasts (Brembilla et al., 2013; Vettori et al., 2020). Conversely, another study showed an inverse correlation between IL-17 A levels and disease activity, having a protective effect in SSc. IL-17 A inhibits fibroblast-to-myofibroblast transformation via transforming growth factor β (TGF-β), hindering collagen production by SSc fibroblasts *in vitro* (Truchetet et al., 2013). In a study conducted by Chizzolini et al. using 3D organotypic skin analogs, IL-17 A stimulates the expression of pro-inflammatory genes without affecting collagen formation (Dufour et al., 2020). T follicular helper cells (TFH cells), a subset of CD4^+^ T-cell, can localize in B-cell follicles through elevated expression of the chemokine receptor type 5 (CXCR5), thereby promoting B-cell immunoglobulin production (Breitfeld et al., 2000; Schaerli et al., 2000). Furthermore, TFH cells can improve immunoglobulin secretion by secreting IL-21 and stimulating B-cell proliferation. Circulating T follicular helper cells (cTfh cells) also function as TFH cells. Elevated IL-21 levels were observed in the serum of patients with SSc than in healthy individuals. This elevation is correlated with the number of plasmablasts, suggesting that abnormal circulating TFH cell expression in patients with SSc could contribute to B-cell changes. Cultivating TFH cells with their B-cell from patients with SSc stimulates plasma cell development and leads to significant levels of immunoglobulin synthesis *in vitro* (Ricard et al., 2019). Taken together, circulating TFH cells exhibit dysregulation in SSc. The resulting immunological defects generated by cTfh cells foster aberrant B-cell activation and differentiation, primarily through excessive production of IL-21. Histologically, several studies have revealed an association between T lymphocytes and skin fibrosis (Lei et al., 2016; Huang et al., 2020), including the infiltration of TFH-like cells in skin lesions of patients with SSc (Taylor et al., 2018; Maehara et al., 2020; Ly et al., 2021). The frequency of TFH-like cell occurrence in the fibrotic lesions of patients with SSc remains uncertain despite their identification of these lesions. Other T lymphocyte subpopulations, such as Cyclin-CD8^+^ T lymphocytes, have been shown to be responsible for the skin tissue fibrotic process (Francois et al., 2013; Dumoitier et al., 2017; Li, 2018; Maehara et al., 2020).

**FIGURE 1:**
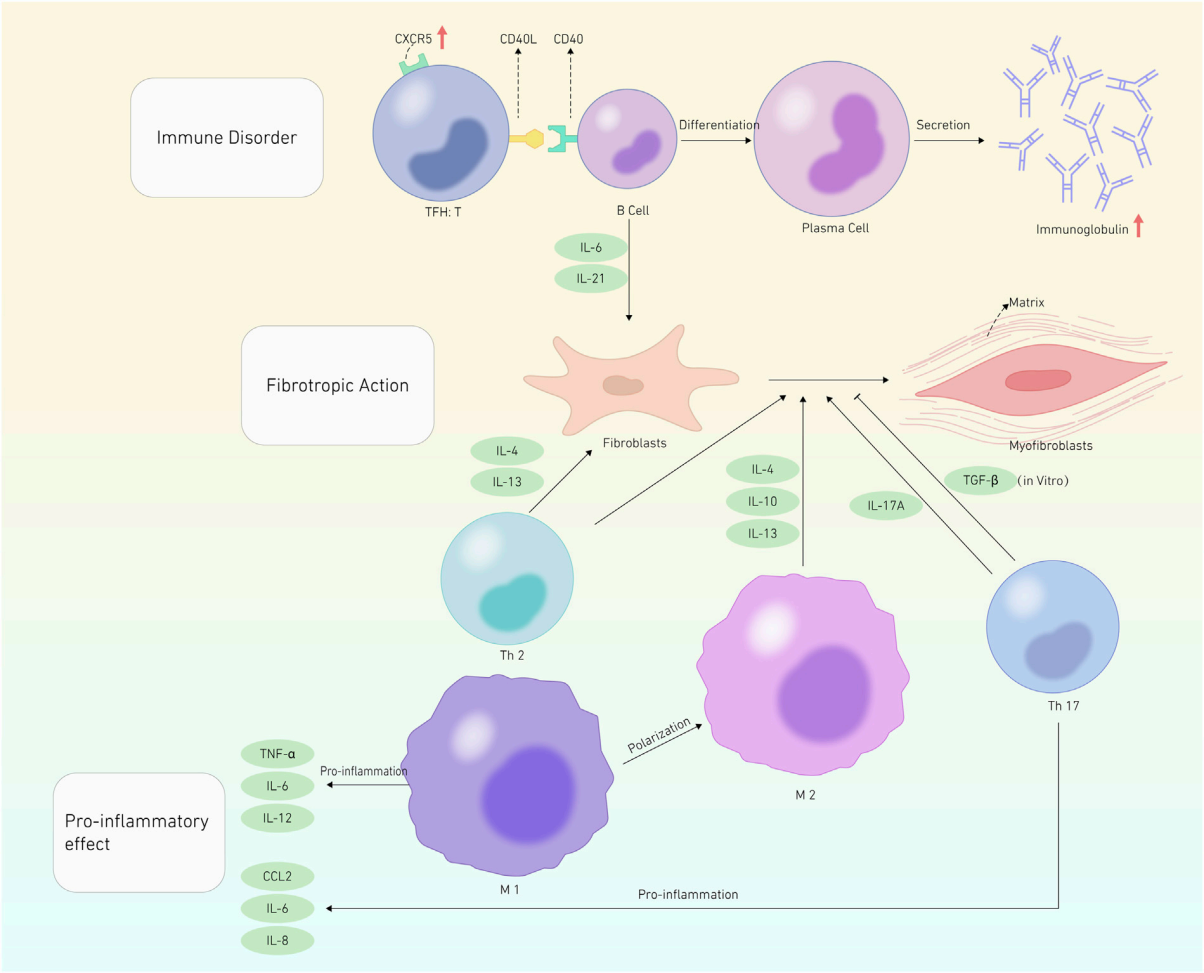
Pathogenesis of SSc. This graphic depicts systemic sclerosis pathogenesis in terms of Immune disoder, Fibrotropic action, and Pro-inflammatory effects. The red cut head represents an increase in the substance’s quantity. The promotion effect is represented by a black shear head, whereas the inhibitory effect is represented by a flat shear head.

Furthermore, macrophages are crucial in scleroderma (Christmann and Lafyatis, 2010). Monocytes enter tissues and, depending on their surroundings, differentiate into macrophages. M1 macrophages phagocytose foreign pathogens, releasing pro-inflammatory molecules, including tumor necrosis factor α, IL-6, and IL-12, activating other immune cells, and triggering the inflammatory response. Conversely, M2 macrophages reduce the inflammatory response by generating interleukin 4, 10, and 13 to repair and remodel tissues, ultimately promoting fibrosis (Figure 1). Therefore, M1 polarization to the M2 type is one of the main causes of M1/M2 macrophage disproportion, leading to SSc fibrosis (Christmann and Lafyatis, 2010). In summary, macrophages trigger the immune system through various pathways, releasing pro-inflammatory and profibrotic mediators. This process spurs fibroblast activation into myofibroblasts, thereby producing large amounts of extracellular matrix, ultimately causing fibrosis.

## 4 MSCs and their exosomes in SSc

### 4.1 MSCs and their exosomes in SSc-associated interstitial lung disease

Interstitial lung disease (ILD) is one of the most common manifestations of SSc. Currently, SSc-associated ILD has a mere 10-year survival rate of only 40%–50% and is the leading cause of SSc-related death (Kawano and Chung, 2021).

#### 4.1.1 Pathogenesis of SSc-associated ILD

Collagen is the main component of the extracellular matrix in lung tissue. It is crucial in maintaining the structural integrity of lung tissue. The occurrence of pulmonary interstitial fibrosis is ultimately caused by abnormal extracellular matrix metabolism, which promotes increased fibrotic factor production or relatively insufficient antifibrotic factor production, culminating in the progression of pulmonary fibrosis. Alveolitis frequently occurs in the early stages of pulmonary fibrosis. It is marked by inflammation in the alveoli and the accumulation of serous fluid and cellular components. The inflammatory process is spurred by numerous monocytes, lymphocytes, plasma cells, alveolar macrophages, and other inflammatory cells infiltrating the pulmonary interstitium. However, the integrity of the alveolar structure remains unaffected. During the advanced stages, the alveoli are replaced by firm collagen, leading to the destruction of the alveolar walls. This process culminates in dilated honeycomb-shaped spaces within the lungs, all of which are pathological changes (Li et al., 2018).

The pathogenesis of pulmonary interstitial fibrosis induced by SSc remains unclear. However, it is established that an abnormal repair process is initiated upon recurrent minor injuries to the alveolar epithelium. This process occurs when immune cells attack the lungs, triggering the epithelial-mesenchymal transition. Consequently, fibroblasts are stimulated and secrete enormous amounts of extracellular matrix. This process promotes the production of cytokines, such as TGF-β and IL-1, by activating the nuclear factor kB and mitogen-activated protein kinase (MAPK) pathways. The cytokines, via autocrine and paracrine effects, mediate the movement of inflammatory cells, including macrophages, toward the stimulated site and promote the proliferation and differentiation of fibroblastic cells (Li et al., 2018). In contrast, RNA-binding protein 7 exhibited increased levels in injured lung epithelial cells in a mouse bleomycin-induced pulmonary fibrosis model. It then interfered with the repair and regeneration process by promoting apoptosis. Moreover, it triggered the degradation of nuclear-enriched abundant transcript 1, resulting in the apoptosis of epithelial cells. This sequence of events subsequently stimulated the production of chemokine (C-X-C motif) ligand 12 (CXCL 12). This led to the recruitment of fibrotic monocyte populations to the damaged areas by segregated-nucleus-containing atypical monocytes (SatM), thereby promoting pulmonary fibrosis (Fukushima and Akira, 2021). Furthermore, activated leukocyte cell adhesion molecules, specifically CD166 (ALCAM; CD166), can block TGF-β and extracellular signal-regulated protein kinase (ERK) signaling pathways. This consequently curtails TGF-β and ERK signaling pathway activation within lung tissues, reducing apoptosis, inhibiting lung tissue repair, and escalating lung fibrosis (Kim et al., 2022).

#### 4.1.2 Mechanism and efficacy of MSCs and their exosomes in treating SSc-ILD

MSCs can induce immunosuppression by bolstering the regulatory T-cell (Treg cells) and IL-10-secreting regulatory B-cell (IL-10^+^ Breg cells) levels. In addition, MSCs contribute to tissue repair by secreting numerous growth factors (Cahill et al., 2016; Li et al., 2017; Tseng and Hoon, 2021). Furthermore, MSCs can use intracellular channels or extracellular vesicles (EVs), mediated by gap junction proteins (EVs), to facilitate the transport of mitochondria from MSCs to impaired cells. This process restores ATP accumulation and function (Morrison et al., 2017; Paliwal et al., 2018). MSCs can mitigate inflammation by secreting inflammatory factors such as IL-4 and prostaglandin E2 (PGE-2) (Shirjang et al., 2017; Harrell et al., 2020). When intercellular contact molecules or soluble factors secreted by MSCs (such as CD45, CD44, CD86, and CD163) interact with T-cell, they can inhibit their maturation. Moreover, these soluble factors can diminish dendritic cell activation and proliferation while inhibiting natural killer cell cytotoxicity (Ni et al., 2018; de Castro et al., 2019; He et al., 2020). MSCs and their exosomes also exhibit antifibrotic attributes by reducing the amount of profibrotic M2 phenotype macrophages (Willis et al., 2018; Luo et al., 2019). Furthermore, they directly counteract fibrotic processes by regulating the ratio of metalloproteinases/tissue inhibitors of metalloproteinases (Xu et al., 2017; Chu et al., 2019). Finally, MSCs can hinder the epithelial-mesenchymal transition, promoting angiogenesis and alveolar repair.

Numerous animal studies have shown that MSCs are effective in treating pulmonary fibrosis (Yang et al., 2021). According to Pereira-Simon et al., intervention in a bleomycin-induced pulmonary fibrosis model revealed that adipose-derived MSCs (AD-MSCs) and umbilical cord-derived MSCs (UC-MSCs) had a palliative impact on pulmonary fibrosis (Periera-Simon et al., 2021). Therefore, this suggests that MSCs from various sources have a therapeutic effect on pulmonary fibrosis. Furthermore, Tashiro et al. investigated the differences between young and old male mice through intratracheal injections of AD-MSCs following bleomycin administration. They found that mice treated with bleomycin from young donor AD-MSCs exhibited significantly lower pulmonary fibrosis and other inflammatory markers than those treated with bleomycin from older donors (Caplan, 2017). This highlights the fact that donor-related factors influence the efficacy of MSCs. In 2017, Zhang et al. conducted a clinical experiment where UC-MSCs were injected into 14 patients with diffuse cutaneous SSc (injection site not reported). They found decreased skin thickness, improved lung function, and adverse reactions of upper respiratory tract infection and diarrhea (Zhang et al., 2017). Furthermore, Zhang et al. found that following intravenous administration of allogeneic UC-MSCs (1 × 10^6^ cells/kg) to patients with interstitial lung disease, lung function, and CT images displayed improvement in all three patients after undergoing 12 months of comprehensive treatment (Ferreli et al., 2017). Farge et al. found that after a single intravenous infusion of allogeneic bone marrow-derived MSCs (BM-MSCs) to 20 patients, their pulmonary function remained stable after 1 year of follow-up. These findings indicate that MSCs have exhibited safe usage and minimal adverse reactions in clinical scenarios, thus offering a promising therapeutic outlook for SSc-ILD.

MSCs serve as one source of exosomes for treating SSc-associated pulmonary fibrosis. In a previous study, we discovered that exosome-derived MSCs can affect lung fibrosis and control cytokine expression linked to this condition. Mansouri et al. found that BM-MSC-derived exosomes significantly reduced bleomycin-induced pulmonary fibrosis 7 days after creating an animal model. These exosomes also altered the inflammatory response of lung tissue. In addition, they found that prior administration of BM-MSC-derived exosomes before bone marrow mononuclear cell transplantation prevented pulmonary fibrosis (Mansouri et al., 2019). Furthermore, UC-MSC-derived exosomes can reduce collagen deposition in lung tissue and alleviate bleomycin-induced pulmonary fibrosis by inhibiting the epithelial-mesenchymal transition process. This process is activated by TGF-β1/mothers against decapentaplegic homolog 2/3 (Smad2/3) signaling pathway (Yang J. et al., 2020).

### 4.2 Research advancement in MSCs and their exosomes in treating SSc-associated skin fibrosis

With modern advancements in science and technology, MSC transplantation provides a promising therapeutic option for treating skin fibrosis in SSc. Results from animal experimental studies revealed that MSCs were effective in treating skin fibrosis in an SSc-mouse model. MSC also reduced collagen synthesis and collagen-Iα1, collagen-Iα2, fibronectin 1, and α-smooth muscle actin gene expression levels. These combined effects underscore their antifibrotic role. In addition, MSC treatment decreased TGF-β, interferon-γ, IL-10, IL-1b, and IL-6 mRNAs expression, indicating the anti-inflammatory effect of MSCs (Yu et al., 2022). The National Institutes of Health (NIH) published a study in 2017 showing that subcutaneous injection of AD-MSCs reduced skin fibrosis in mice and significantly decreased the amount of hydroxyproline in subcutaneous tissue. UC-MSCs have garnered more attention than BM-MSCs due to their simpler collection process, ethical considerations, and safety factors (Arutyunyan et al., 2016). Besides sharing characteristics with BM-MSCs, our previous research highlighted that UC-MSCs also exhibited various immunomodulatory molecules, including TGF-β1, indoleamine2,3-dioxygenase (IDO), tumor necrosis factor-alpha stimulated gene-6, and PGE-2 (Sabapathy et al., 2014). Based on the specific benefits of UC-MSCs, Yang, Yuan, et al. (2020) revealed that UC-MSCs suppressed the inflammatory response at the lesions and inhibited bleomycin-induced collagen production at the lesions, thus lowering fibrosis (Rad et al., 2019; Yang Y. et al., 2020). The animal trials conducted previously demonstrated that MSCs can markedly improve skin fibrosis, a breakthrough that opens up a new avenue for using MSCs in treating SSc-related skin fibrosis, presenting a novel treatment alternative. These findings further imply that MSCs can ameliorate the fibrotic condition of the skin, thereby resulting in skin regeneration. A recent study revealed that UC-MSC-derived exosomes reduced bleomycin-induced skin fibrosis for at least 3 weeks (Yu et al., 2022). In addition, we discovered that UC-MSC-derived exosomes inhibited the conversion of vascular endothelial cells to mesenchymal cells (Alexander and Greco, 2022). In a clinical trial, Khanna et al. (2018) injected AD-MSCs subcutaneously into all fingers of patients with impaired function, including diffuse and localized sclerosis cases. The findings of the study revealed several improvements: the Cochin Hand Function Score improved, the Range of Motion Score decreased, and the Sheffield Hand Assessment Questionnaire score indicated an increase. Additionally, the EQ(5D) 5-level assessment showed improvement in patients with diffuse, and the global assessment of SSc activity for diffuse patients improved compared with the corresponding parameters before the intervention. However, the intervention was associated with sequelae, such as upper respiratory tract infection, arthralgia, cellulitis, limb pain, hypoesthesia, and other related symptoms (Magalon et al., 2019). Guiducci et al. found that intravenous infusion of MSCs into patients with SSc led to decreased necrotic skin area, microscopic tubular cell populations on skin section analysis, and a robust expression of angiogenic factors (Table 1) (Guiducci et al., 2010). Therefore, MSCs are considered safe for studying SSc in humans; however, their efficacy requires further confirmation.

**TABLE 1:** Summary of current clinical trails in MSCs and MSC-EVs on SSc.

NO	Trail ID	Disease	Title	Inclusion criteria	No. of Patients	Cell type	Route	No of cells per infusion	No. of infusions	Safety outcome	Efficacy outcome	Phase	Locations
1	NCT	SSc	Safety of Cultured Allogeneic Adult Umbilical Cord Derived Mesenchymal Stem Cell IV Infusion for Systemic Sclerosis	Diagnosis of Systemic Sclerosis	20	Allogeneic	IV		Single intravenous infusion of 100 million cells	No results posted	No results posted	I	Antigua and Barbuda, Argentina, Mexico
	05016804			Understanding and willingness document to sign a written informed consent		UC-MSCs							
2	NCT	SSc	Infusion of AllMesenchymal Stem Cells in Patients With Diffuse Cutaneous Systemic Sclerosis With Refractory Pulmonary Involvement	Age >18 years and <65 years		Mesenchymal Stem Cells from Wharton s jelly	IV	2 × 10 ^ 6 mesenchymal cells per kilogram of patient weight		No results posted	No results posted	Ⅰ	Universidad de la Sabana, Chía, Chia, Colombia
	04432545			Established diagnosis of systemic sclerosis according to the criteria of the American College of Rheumatology									
3	NCT	SSc	Allogeneic Mesenchymal Stem Cells Transplantation for Systemic Sclerosis (SSc)	Fulfilled the American College of Rheumatology (former American Rheumatism Association - ARA) for SSc patients	20	Allogeneic Mesenchymal Stem Cells	IV	10 ^ 6 cells/kg body weight		No results posted	No results posted	I/II	China, Jiangsu
	00962923												
4	NCT	SSc	Treatment of Refractory Sever Systemic Scleroderma by Injection of Allogeneic Mesenchymal Stem Cells (MSC) (Guiducci et al., 2010)	Age >18 years and <70 years	20	Allogeneic	IV			There were no instances of treatment-emergent adverse events	Regression of skin sclerosis after early infusion, and stable pulmonary function until 1 year post infusion	I/II	Saint-Louis Hospital Paris, France
	02213705			Established diagnosis of systemic sclerosis according to the criteria of the American College of Rheumatology		-MSCs							
5	NCT	SSc	ADMSCs for the Treatment of Systemic Sclerosis	2013 American college of Rheumatology/European League Against Rheumatism Criteria for the classification of systemic sclerosis	7	Inject autologous Stromal vascular fraction	IV			No results posted	No results posted	Not Applicable	Korea, Republic of
	02975960												
6	NCT	SSc	Treatment With Human Umbilical Cord-derived Mesenchymal Stromal Cells in Systemic Sclerosis (CARE-SSc)	SSc according to American College of Rheumatology/European League Against Rheumatism (ACR/EULAR) 2103 classification criteria for systemic sclerosis	18	UC-MSCs	IV	1 million UCMSC/kg suspended in 50 ml		No results posted	No results posted	I/II	China
	04356287												

### 4.3 MSCs and their exosomes in SSc-related vascular injury

#### 4.3.1 Pathogenesis of SSc-related vascular injury

Vascular injury represents an early event in SSc pathogenesis, significantly influencing the onset of pulmonary hypertension, finger-end ulcers, and Raynaud’s phenomenon (Kahaleh, 2004; Abraham and Distler, 2007; Cipriani et al., 2011; Matucci-Cerinic et al., 2013; Asano and Sato, 2015; Odonwodo et al., 2023). However, the underlying cause of chronic inflammation is the combination of autoimmunity, vascular injury, and fibrosis. Among these factors, vascular lesions and the autoimmune inflammatory response serve as the pathological foundation of the disease and are two key drivers of its progression (Abraham and Varga, 2005; Senecal et al., 2020; Shimizu et al., 2022).

In early SSc stages, vascular remodeling occurs, characterized by intimal hyperplasia, capillary dilatation, and microvascular intracellular sugar aggregation following endothelial cell injury. Subsequently, avascular zones develop due to damage to capillaries and small arteries (Koch and Distler, 2007; Hughes and Herrick, 2019). Larger arteries can become occluded and form thrombi due to endothelial proliferation and fibroproliferation, fibrin deposition, and smooth muscle cell hypertrophy (Koch and Distler, 2007; Kavian and Batteux, 2015; Hughes and Herrick, 2019). One common clinical SSc symptom, Reynold’s disease, is characterized by alternating episodes of ischemia-reperfusion and persistent arterial vasospasm, with elevated junctional adhesion molecule (JAM) expression (Koch and Distler, 2007). In a recent study, we discovered that JAMs were substantially upregulated in the vascular endothelium, leading to increased binding of macrophages and platelets to the endothelium. Endothelial cell damage is caused by increased superoxide anion generation by neutrophils and platelets (Hughes and Herrick, 2019). Therefore, in the early stages of SSc, endothelial cell destruction might be accompanied by impaired neurological function, resulting in vascular damage (Kahaleh, 2004; Abraham and Distler, 2007; Hughes and Herrick, 2019). Thus, while the precise pathophysiology of endothelial cell damage remains unknown, autoimmunity appears to be connected with the production of cytokines and adhesion molecules by epithelial cells, eventually culminating in endothelial cell apoptosis (Lunardi et al., 2000; Ahmed et al., 2006; Mulligan-Kehoe and Simons, 2008; Hughes and Herrick, 2019).

#### 4.3.2 Mechanism and efficacy of MSCs and their exosomes in treating SSc-related vascular injury

Recent research has revealed that local or systemic autologous stem cell transplantation in SSc can be used to efficiently treat peripheral vascular lesions such as finger ulcers and limb necrosis (Nevskaya et al., 2009; Ishigatsubo et al., 2010; Yun, 2011). For example, in SSc patients with acute limb gangrene, the peripheral vascular network can be regenerated through intravenous injection, culture, and amplification of autologous bone marrow mesenchymal stem cells, consistent with findings from previous studies (Yun, 2011). Guiducci S. et al. Discovered that MSCs can secrete pro-angiogenic factors such as TGF-β1, which promotes vascular regeneration and allows pericytes, vascular smooth muscle cells, and other cells to play a significant role in the vascular regeneration process. Concurrently, MSCs drive their differentiation into pericytes and smooth muscle cells during neovascularization, thereby enhancing neovascularization stabilization. However, whether SSc-MSCs can be guided effectively to develop into epithelial cells within the vessel wall remains to be seen. Komaki et al. used a mouse ear ischemia injury model to investigate the pro-neovascularization effect of human-term placental tissue-derived MSC exosomes (PlaMSC-exos). They discovered that the pro-neovascularization effect was mediated by exosomes released from placental tissue-derived MSCs. Therefore, PlaMSC-exo is being considered as a potential treatment strategy (Komaki et al., 2017).

### 4.4 Research progress on MSCs and their exosomes in other organ involvement associated with SSc

#### 4.4.1 Pathogenesis of gastrointestinal involvement in systemic sclerosis

Gastrointestinal (GI) tract involvement is prevalent in 90% of patients with SSc, marked by lesions spanning from the oral cavity to the anus, causing symptoms such as GI dysmotility, esophagitis, gastritis, gastroesophageal reflux, and mucosal ulcers. In severe cases, this can lead to challenges in swallowing and nutrient malabsorption. It significantly affects the quality of life of patients with SSc (Gyger and Baron, 2012). Therefore, comprehending the underlying mechanism of these diseases is essential for developing an appropriate treatment program for GI diseases. Two main theories exist regarding the GI tract lesion mechanism (Emmanuel, 2016). The first is related to autonomic nerve axonal damage (i.e., GI motility dysfunction caused by sympathetic overactivity). The second is associated with vascular and autoimmune phenomena (i.e., progressive fibrotic changes in the GI tract due to impaired collagen and other extracellular matrix component deposition). However, regardless of the explanation, GI involvement results from a chain of responses rather than a single component.

#### 4.4.2 Pathogenesis of renal involvement in systemic sclerosis

Scleroderma renal crisis (SRC), a primary cause of death in diffuse scleroderma, is frequently associated with SSc. SRC is estimated to affect 5%–10% of patients with systemic sclerosis (Woodworth et al., 2016; Hudson et al., 2021). Most patients experience malignant hypertension and increased renal impairment. SRC is linked to the renin-angiotensin-aldosterone system activation, upregulation of endothelin-B receptors, and activation of anti-endothelial cell antibodies. These factors cause renal vasoconstriction and ischemia (Scheen et al., 2023).

#### 4.4.3 Pathogenesis of cardiac involvement in systemic sclerosis

In diffuse cutaneous SSc, the heart is among the initial organs to be affected in patients with SSc. The most prevalent symptoms include myocardial fibrosis, coronary artery disease, pericarditis, and heart failure. Heart disease accounts for 27.2% of SSc-related fatalities (Wangkaew et al., 2017). Therefore, early detection is crucial. Furthermore, since the heart of patients with SSc may exhibit transitory vasospasm and functional changes in the early stages of the disease, distinguishing symptoms are usually absent in the early stage. The fibrotic changes in the coronary arteries result in permanent damage to heart function resulting in systemic blood circulation anomalies (Kahan et al., 1985).

#### 4.4.4 Pathogenesis of musculoskeletal involvement in systemic sclerosis

Musculoskeletal involvement is the leading cause of reduced mobility and disability in patients with SSc. Arthralgia is the most common symptom, and poor prognosis is linked to tendon friction. Recent research has revealed a link between elevated blood YKL-40 levels and chondrocyte and/or fibroblast activity in patients with SSc (Lorand et al., 2014).

#### 4.4.5 Mesenchymal stem cells and their exosomes for various organs related to systemic sclerosis

Inflammation and the autoimmune response play a pivotal role in organ damage in patients with SSc. MSCs and their exosomes reduce inflammation and fibrosis in the heart, kidney, digestive tract, and musculoskeletal system by modulating the immune system and reducing the release of inflammatory mediators and immune cell activation. Simultaneously, a sufficient amount of pro-angiogenic molecules, such as vascular endothelial growth factor, and basic fibroblast growth factor is released. These molecules stimulate angiogenesis, repair, and enhance blood circulation and oxygenation. Furthermore, MSCs and their exosomes can prevent the production of fibrosis-related cytokines and collagen, thereby lowering the extent of organ fibrosis. Finally, their high regenerative potential can facilitate tissue healing and functional recovery in damaged areas.

## 5 Brief summary and outlook

In summary, MSCs and exosomes have been proven to improve lung function, reduce cutaneous fibrosis, promote neovascularization, and mitigate fibrosis in the heart, kidneys, digestive tract, and musculoskeletal system to varying degrees. However, it is undeniable that the research and utilization of MSCs and their exosomes have certain limitations due to their early stage. The limitations are as follows: the aging and functional degradation of MSCs, distribution and local differentiation *in vivo*, and the potential tumorigenic risk, which have hindered the development of MSCs in clinical research and application to a certain extent, whereas exosomes, due to their low extracellular yield, high demand, and limited therapeutic capacity, have prompted researchers to urgently search for methods that can improve the therapeutic effects. Furthermore, to optimize the therapeutic efficacy of MSCs and exosomes, it is essential to conduct comprehensive research on dosage, frequency, administration methods, timing (early or late stage of the disease), and donor status (age and health). Additionally, MSCs hold potential for diverse applications in various sectors, such as their chemical modification for use as drug carriers for tracer effects. In conclusion, while MSCs and their exosomes have favorable and extensive benefits in treating SSc, further clinical trials are required to address some potential concerns.

## Figures and Tables

**Table:** 

**SSc**	Systemic sclerosis
**MSCs**	Mesenchymal stem cells
**AHSCT**	Autologous haematopoietic stem cell transplantation
**FVC**	Forced Vital Capacity
**Th cells**	Helper T-cell
**CCL2**	Chemokine ligand 2
**TGF-β**	Transforming growth factor β
**TFH cells**	T follicular helper cells
**CXCR5**	Chemokine receptor type 5
**cTfh cells**	Circulating T follicular helper cells
**TNF-α**	Tumor necrosis factor α
**ILD**	Interstitial lung disease
**MAPK**	Mitogen-activated protein kinase
**NF-κB**	Nuclear factor κB
**RBM7**	RNA-binding protein 7
**CXCL 12**	Chemokine (C-X-C motif) ligand 12
**NEAT1**	Nuclear enriched abundant transcript 1
**SatM**	Segregated-nucleus-containing atypical monocytes
**ALCAM; CD166**	activated leukocyte cell adhesion molecules; CD166
**ERK**	Extracellular signal-regulated protein kinase
**Treg cells**	regulatory T-cell
**IL-10+ Breg cells**	IL-10-secreting regulatory B-cell
**EVs**	Extracellular vesicles
**PGE-2**	Prostaglandin E2
**AD-MSCs**	Adipose-derived MSCs
**UC-MSCs**	Umbilical cord-derived MSCs
**BM-MSCs**	Bone marrow-derived MSCs
**SSc-ILD**	Systemic sclerosis associated interstitial lung disease
**Smad2/3**	Mothers against decapentaplegic homolog 2/3
**NIH**	National Institutes of Health
**IDO**	indoleamine2,3-dioxygenase
**TSG-6**	tumor necrosis factor alpha stimulated gene-6
**CHFS**	Cochin Hand Function Score
**RCS**	the Range of Motion Score
**SHAQ**	the Sheffield Hand Assessment Questionnaire
**JAM**	Junctional adhesion molecule
**PlaMSCs**	Placental tissue-derived MSCs
**PlaMSC-exos**	Placental tissue-derived MSC exosomes
